# EGFR Alterations Influence the Cetuximab Treatment Response and c-MET Tyrosine-Kinase Inhibitor Sensitivity in Experimental Head and Neck Squamous Cell Carcinomas

**DOI:** 10.3389/pore.2021.620256

**Published:** 2021-05-03

**Authors:** Györgyi A. Nelhűbel, Mihály Cserepes, Balázs Szabó, Dóra Türk, Adél Kárpáti, István Kenessey, Erzsébet Rásó, Tamás Barbai, Zita Hegedűs, Viktória László, Bálint Szokol, Judit Dobos, László Őrfi, József Tóvári

**Affiliations:** ^1^ Department of Experimental Pharmacology, National Institute of Oncology, Budapest, Hungary; ^2^ 2^nd^ Department of Pediatrics, Semmelweis University, Budapest, Hungary; ^3^ Department of Otolaryngology and Head and Neck Surgery, Semmelweis University, Budapest, Hungary; ^4^ 2^nd^ Institute of Pathology, Semmelweis University, Budapest, Hungary; ^5^ Hungarian Cancer Registry, National Institute of Oncology, Budapest, Hungary; ^6^ Department of Thoracic Surgery, Medical University of Vienna, Vienna, Austria; ^7^ Department of Tumor Biology, National Korányi Institute of Pulmonology, Budapest, Hungary; ^8^ Vichem Chemie Ltd., Budapest, Hungary

**Keywords:** EGFR, c-Met, HNSCC, R521K, targeted tumor therapy

## Abstract

**Background:** Anti-EGFR antibody therapy is still one of the clinical choices in head and neck squamous cell carcinoma (HNSCC) patients, but the emergence of cetuximab resistance questioned its effectiveness and reduced its applicability. Although several possible reasons of resistance against the antibody treatment and alternative therapeutic proposals have been described (EGFR alterations, activation of other signaling pathways), there is no method to predict the effectiveness of anti-EGFR antibody treatments and to suggest novel therapeutics. Our study investigated the effect of EGFR R521K alteration on efficiency of cetuximab therapy of HNSCC cell lines and tried to find alternative therapeutic approaches against the resistant cells.

**Methods:** After genetic characterization of HNSCC cells, we chose one wild type and one R521K+ cell line for *in vitro* proliferation and apoptosis tests, and *in vivo* animal models using different therapeutic agents.

**Results:** Although the cetuximab treatment affected EGFR signalization in both cells, it did not alter *in vitro* cell proliferation or apoptosis. *In vivo* cetuximab therapy was also ineffective on R521K harboring tumor xenografts, while blocked the tumor growth of EGFR-wild type xenografts. Interestingly, the cetuximab-resistant R521K tumors were successfully treated with c-MET tyrosine kinase inhibitor SU11274.

**Conclusion:** Our results suggest that HNSCC cell line expressing the R521K mutant form of EGFR does not respond well to cetuximab treatment *in vitro* or *in vivo*, but hopefully might be targeted by c-MET tyrosine kinase inhibitor treatment.

## Introduction

Over 600,000 head and neck squamous cell carcinoma (HNSCC) patients are diagnosed annually worldwide [1]. In Hungary, they represent the third most abundant type of malignant tumors among men [2]. Patients with early (I-II) stage localized HNSCC undergo radiation or surgery as monotherapy, while patients with advanced tumors (stage III-IV) are treated by combination therapy [3]. The 5-years survival of these patients still remains less than 50% [4,5] therefore finding new therapeutic regimes is an urgent need for clinical practice.

As in many epithelial tumor types, EGFR protein is overexpressed in HNSCC, and increased EGFR gene copy number is frequently described in this region as well [6–9]. Due to its molecular characteristics EGF receptor seemed to be a promising target in the treatment of HNSCC.

At present, there are two different ways to inhibit signaling pathway via EGF receptor. First, the use of anti-EGFR monoclonal antibodies (for example cetuximab, C225, Erbitux®) which bind to the extracellular ligand binding domain of the receptor. Second, using low molecular weight tyrosine kinase inhibitors (for example gefitinib, ZD 1839, Iressa®, or erlotinib, OSI 774, Tarceva®) targeting the intracellular tyrosine kinase domain of the receptor.

Cetuximab has been extensively studied in preclinical models and clinical experiments as well. As former studies have shown [10,11], nowadays, cetuximab is indicated to be used in two stages of the treatment of HNSCC: it was approved by FDA for the treatment of locally or regionally advanced HNSCC in part of chemoradiotherapy, or as monotherapy for patients with relapsed or metastatic tumors, whose platinum-based treatment has formerly failed [12].

The antitumor activity of low molecular weight tyrosine kinase inhibitors (TKIs) has been studied thoroughly in many EGFR overexpressing solid tumors: non-small cell lung cancer (NSCLC), pancreatic cancer, glioblastoma and head and neck cancer. Erlotinib treatment has a significant effect on survival in patients with NSCLC, however, it showed only a modest activity in HNSCC patients [13]. Introducing gefitinib in the therapy of head and neck tumors seemed to be promising based on the molecular characteristics of HNSCCs, nevertheless, in many clinical trials survival benefit and response rate were very low [14,15].

Despite the fact that, based on experimental data, EGFR seemed to be one of the most promising molecular targets in HNSCC, it did not always meet the expectations in clinical practice. Previous results showed that only 10–20% of cancer patients have major clinical responses to EGFR inhibition, either because they barely respond primarily or they acquire resistance during anti-EGFR treatment [16]. Taken together, these data showed that the usage of EGFR inhibition does not necessarily lead to a considerably improved survival rate [17].

The EGFR genotype and phenotype have been reported to have dramatic effect on anti-EGFR therapy efficacy. The classical mutations already used in multiple cancer diagnostics and therapy selection are affecting the intracellular tyrosine kinase domain (exons 19–22). Their role is clear in overcoming TKI therapy efficacy. However, much less is known about the extracellular variations of the receptor, which might confer resistance or hypersensitivity to antibody treatment. In HNSCC, EGFRvIII variant (suffering deletion of exons 2–7), and single nucleotide polymorphism R521K are reported. However, according to our ongoing screening on clinical samples of HNSCC patients, EGFRvIII was not found in more than 1 percent of the patients, therefore was ruled out as possible explanation of different patient response. Importantly, the missense mutation R521K, was quite abundant in our clinical cohort, and, knowing that its position is in the neighborhood of the cetuximab-binding site of the receptor [18], we focused on its possible correlations with *in vitro*, *in vivo* and clinical response to cetuximab-based therapy.

A potent mechanism that may cause resistance to anti-EGFR therapy is activation of alternative receptor tyrosine kinases and signaling pathways (PI3K/AKT, c-Met, Src) [19,20]. The usage of the specific inhibitors of these other kinases in monotherapy or in combination with EGFR inhibition may have increase clinical efficacy in anti-EGFR therapy resistant HNSCC tumors [16,21].

Hepatocyte growth factor (HGF) and its receptor, c-MET are major regulators of cell proliferation, survival, migration and angiogenesis. In tumor cells they contribute to proliferation, apoptosis inhibition, invasion, as well as adhesion. Eventually the HGF-c-MET system confers tumor progression and metastasis. c-MET protein overexpression has been reported in various human epithelial cancer types including HNSCC. Moreover, c-MET has been found in increased amount in lymph node metastases of HNSCC compared with primary tumors [22,23]. This suggests that c-MET has a pivotal role in the development of the metastatic potential of HNSCCs [22].

In the current experimental study, we have investigated the effect of different EGFR inhibitors (cetuximab and erlotinib), the specific c-MET TK inhibitor SU11274 and the RAS protein specific inhibitor zoledronic acid on proliferation and apoptosis of human squamous cell carcinoma cells *in vitro* as well as on the growth and metastasis of HNSCC xenografts *in vivo*, alone and in combination as well.

## Methods

### Cell Lines and Culture Conditions

The PE/CA-PJ15 (oral SCC from a 45-year-old man, ECACC No: 96121230) and PE/CA-PJ41 (oral SCC from a 67-year-old woman, ECACC No: 98020207) human head and neck squamous carcinoma cell lines were obtained from ATCC. Cells were grown in Iscove’s Modified Eagle Medium (IMEM, Sigma-Aldrich, St. Louis, MO), with the addition of 10% fetal bovine serum (Sigma) and 1% penicillin-streptomycin (Sigma) in a 37°C incubator, using 5% CO_2_ concentration.

### Identification of EGFR Extracellular Domain Genetic Alterations and Quantitative Measurement of mRNA Expression of EGFR in HNSCC Cell Lines

Total RNA was isolated from the *in vitro* growing tumor cell cultures using TRIzol® Reagent (Invitrogen, Carlsbad, CA, United Status) and Direct-zol RNA miniprep kit (Zymo research, Irvine, CA), according to the manufacturer’s instructions. Possible DNA contamination was eliminated using Dnase I treatment. For reverse transcription, 1 μl of 10 mM dNTP mix (Finnzyme, Espoo, Finland) and 1 μl of Random primer-oligo dT was added and transcribed 2 μg of the purified genomic RNA. After incubation of the mixture at 70°C for 10 min, 2 μl of 10× M-MLV Reverse transcriptase Buffer (Sigma), 1 μl of M-MLV Reverse transcriptase (200 units/μl, Sigma) enzyme, 0.5 μl RNase Inhibitor (40 units/μl, Promega, Madison, WI 53711, United States) and 6.5 μl nuclease-free water were added. The reactions were incubated at 37°C for 50 min and 85°C for 10 min. Successful reverse transcription was proved by PCR reactions with β-actin primers as a housekeeping gene probe, followed by agarose gel electrophoresis. DNA contamination of the template RNA and nuclease-free water was also controlled every time.

For detecting transcribed EGFR-ECD, serial PCR reactions were carried out with seven different nested primer pairs designed by Array Designer Oligo and cDNA Microarray Design Software (Premier Biosoft International). The primers were optimized to target reference sequence NM_005228.3. The PCR reactions contained 11.5 μl AmpliTaq® Gold 360 MasterMix (Applied Biosystems), 2.5–2.5 μl of the primers (see primers in Table 1), 2 μl of the cDNA template, and 6.5 μl nuclease free water. The reaction program included: 95°C for 10 min, then 38 cycles of 95°C for 1 min, 55°C for 1 min, 72°C for 2 min. Final extension was at 72°C for 5 min. PCR products were separated on a 2% agarose gel and captured with the MULTI GENIUS Bio Imaging System (Syngene, Frederick, MD), recording visual signal of ethidium bromide labeling applied. Transcribed fragments were identified according to the size of the separated PCR products. The proper band was excised and DNA was isolated using the QIAquick Gel Extraction Kit (Qiagen, Valencia, CA, United States). The purified PCR fragments of EGFR-ECD were analyzed by direct sequencing in both directions. BigDye® Terminator v1.1 Cycle Sequencing Kit (Applied Biosystems™–by Life Technologies™) was used for the sequencing, according to the manufacturer’s protocol (the primers were identical to those used at PCR amplification). We purified the sequencing reaction products using BigDye® XTerminator™ Purification Kit (Applied Biosystems™–by Life Technologies™). PCR products were analyzed by a 4-capillary automated sequencer (Applied Biosystems 3130 Genetic Analyzer, Applied Biosystems).

**TABLE 1:** PCR primer list used in EGFR genotyping screen.

Gene	Primer name	Sequence (5′ to 3′)	Length
EGFR extracellular domain screen	EGFR-ECDos1 (190–210)	CCT​GAC​TCC​GTC​CAG​TAT​TGA	392 bp
	EGFR-ECDoa1 (739–758)	TCA​CTG​CTG​ACT​ATG​TCC​CG	
	EGFR-ECDis1 (190–210)	CCT​GAC​TCC​GTC​CAG​TAT​TGA	
	EGFR-ECDia1 (558–582)	GTA​CAT​ATT​TCC​TCT​GAT​GAT​CTG​C	
	EGFR-ECDos2 (309–328)	GAG​TCG​GGC​TCT​GGA​GGA​AA	373 bp
	EGFR-ECDoa2 (861–880)	TGG​TCA​GTT​TCT​GGC​AGT​TCT​C	
	EGFR-ECDis2 (498–517)	GGT​GGC​TGG​TTA​TGT​CCT​CA	
	EGFR-ECDia2 (852–871)	TCT​GGC​AGT​TCT​CCT​CTC​CT	
	EGFR-ECDos3 (643–663)	AAG​GAG​CTG​CCC​ATG​ACA​GAA​AT	376 bp
	EGFR-ECDoa3 (1212–1232)	CAC​TTC​TTA​CAC​TTG​CGG​ACG	
	EGFR-ECDis3 (781–800)	GAC​TTC​CAG​AAC​CAC​CTG​GG	
	EGFR-ECDia3 (1134–1157)	TGA​TCT​GTC​ACC​ACA​TAA​TTA​CGG	
	EGFR-ECDos4 (1061–1080)	CCA​CCA​CGT​ACC​AGA​TGG​AT	388 bp
	EGFR-ECDoa4 (1591–1610)	TCC​TTG​AGG​GAG​CGT​AAT​CC	
	EGFR-ECDis4 (1067–1087)	CGT​ACC​AGA​TGG​ATG​TGA​ACC	
	EGFR-ECDia4 (1434–1455)	CCC​TGT​GAT​TTC​CTT​TAC​GGT​T	
	EGFR-ECDos5 (1334–1353)	CCT​CCA​TCA​GTG​GCG​ATC​TC	364 bp
	EGFR-ECDoa5 (1895–1916)	TGT​ATG​CAC​TCA​GAG​TTC​TCC​A	
	EGFR-ECDis5 (1366–1385)	GTG​GCA​TTT​AGG​GGT​GAC​TC	
	EGFR-ECDia5 (1706–1730)	CCT​CTG​TTG​CTT​ATA​ATT​TTG​GTT​T	
	EGFR-ECDos6 (1608–1632)	GGA​GAT​AAG​TGA​TGG​AGA​TGT​GAT​A	368 bp
	EGFR-ECDoa6 (2139–2158)	TTG​GAC​AGC​CTT​TAA​GAC​CT	
	EGFR-ECDis6 (1621–1645)	GGA​GAT​GTG​ATA​ATT​TCA​GGA​AAC​A	
	EGFR-ECDia6 (1969–1989)	CTG​GAT​ACA​GTT​GTC​TGG​TCC	
	EGFR-ECDos7 (1759–1778)	GTC​TGC​CAT​GCC​TTG​TGC​TC	343 bp
	EGFR-ECDoa7 (2319–2339)	AGC​TTC​TCC​ACT​GGG​TGT​AAG​A	
	EGFR-ECDis7 (1911–1930)	CAT​ACA​GTG​CCA​CCC​AGA​GT	
	EGFR-ECDia7 (2235–2254)	TTC​GCA​TGA​AGA​GGC​CGA​TC	
β-actin	βS1	TCT​GGC​ACC​ACA​CCT​TCT​AC	387 bp
	βA4	CTC​CTT​AAT​GTC​ACG​CAC​GAT​TTC	
EGFRvIII	EGFRvIIIS	AGT​CGG​GCT​CTG​GAG​GAA​A	95 bp/896 bp
	EGFRvIIIA	TCC​TCC​ATC​TCA​TAG​CTG​TC	
EGFRwt	EGFRwtS	TAC​CTA​TGT​GCA​GAG​GAA​TTA​TGA​TCT​TT	89 bp
	EGFRwtA	CCA​CTG​TGT​TGA​GGG​CAA​TG	

Primer pairs designed by Array Designer Oligo and cDNA Microarray Design Software—Premier Biosoft International.

For the detection of the expressed EGFRvIII isoform, a variant-specific PCR reaction (primers shown in Table 1) was used. Besides HNSCC cell lines, we used U87vIII glioblastoma cell line (Sigma)—transduced with EGFR vIII isoform—sample as a positive control. The reactions were processed in 20 μl volume containing 10 μl 2 × GoTaq Green PCR mix, 1 μl forward and reverse primer, 500 ng cDNA template, and nuclease-free water was added to reach the final volume. The PCR protocol included activation phase at 94°C for 4 min, 35 polymerization cycles (94°C for 30 s—60°C for 30 s—72°C for 1 min), and a final elongation phase at 72°C for 10 min. The PCR products were then separated in 1% agarose gel horizontal electrophoresis system using EcoSafe (Pacific Image Electronics, Taiwan) gel stain at a dilution of 1:20,000 in order to visualize DNA for photographing.

In parallel, we measured the quantity of wild type version of EGFR (primer is3/ia3) by real-time PCR reaction measurements. Reaction mixtures contained 12.5 μl ABsolute™ QPCR SYBR® Green Mix (Thermo Fisher Scientific Carlsbad, CA), 0.5–0.5 μl of each primer (reaching primer concentration of 200 nM) and 11.5 μl of the cDNA template. The cycling conditions (LightCycler 480 II, Roche Diagnostics, Basel, Switzerland) included 3 minutes of DNA polymerase activation at 95°C, and 40 cycles of the following steps: 95°C for 30 s, 55°C for 30 s, 72°C for 60 s. Starting quantities were defined on the basis of standard dilution series (1×-625×). cDNA of PC3 human prostate carcinoma cell line was used as control template.

The cell lines were also examined for KRAS, NRAS, EGFR tyrosine kinase domain genotypes and HPV (see primers in Supplementary Table S1).

### Immunocytochemistry of HNSCC Cells

To detect EGFR, c-MET or the activated receptor (phospho-EGFR, and phospho-c-MET) proteins, HNSCC cells were fixed in 4% paraformaldehyde for 10 minutes, permeabilized using 0.1% Triton X-100 (Sigma) PBS for 1 min. After washing, we blocked non-specific reactions with 1% bovine serum albumin (BSA; Sigma) for 30 min at room temperature. Samples were then incubated with the following primary antibodies: EGF Receptor (D38B1) XP® Rabbit mAb #4267, Phospho-EGF Receptor (Tyr1068) (D7A5) XP® Rabbit mAb #3777, Met (D1C2) XP® Rabbit mAb #8198, Phospho-Met (Tyr1234/1235) (D26) XP® Rabbit mAb #3077. All primary antibodies were obtained from Cell Signaling Technology (Danvers, MA). We incubated the samples at 37°C for one hour, followed by washing in PBS for 3 × 10 min, and incubated with Alexa-488 conjugated goat anti-rabbit IgG (H + L) antibody (Thermo Fisher Scientific) for 45 min at 37°C (dilution 1:100). Negative controls were prepared by replacing the primary antibody with isotype-matched non-immune IgG (Sigma). Cell nuclei were stained with 1 μg/ml Hoechst 33,342 (Sigma) for 2 min at room temperature. Slides were covered with Prolong Gold antifade reagent (Thermo Fisher Scientific), and cells were photographed using fluorescent microscopy (Leica DM IL LED Microscope with mercury lamp and Leica DFC345 FX camera). All images were produced with the same exposure setup for the same channel.

### EGFR, RAS and c-MET Inhibitors

EGFR-specific humanized monoclonal antibody (cetuximab, IMC-225, Erbitux®; Merck) was used at concentrations of 10 and 100 μg/ml in RPMI for *in vitro*, and at 400 mg/m^2^ loading dose followed by 250 mg/m^2^ sustaining dose in physiological salt solution weekly for *in vivo* studies.

EGFR specific small molecule TKI erlotinib (OSI 774, Tarceva®; Roche) was used at concentrations of 5 and 25 μM for *in vitro* studies. In animal experiments, SCID mice were treated at human equivalent dose daily with erlotinib at 2 mg/kg.

In our setup, we also used the RAS inhibitor zoledronic acid (Zometa; Novartis Europharm), at concentrations of 10 and 50 μM in cell proliferation assays, and 50 μg/kg for *in vivo* experiments.

Cetuximab, erlotinib, and zoledronic acid were used in aqueous stock solutions, and diluted in cell culture medium for *in vitro* assays, or physiological saline with *in vivo* experiments.

The c-MET-specific inhibitor [24] SU11274 [(3Z)-N-(3-chlorophenyl)-3-({3,5-dimethyl-4-[(4-methylpiperazin-1-yl)carbonyl]-1H-pyrrol-2-yl}methylene)-N-methyl-2-oxo-2,3-dihydro-1H-indole-5-sulfonamid] (Pfizer Inc, San Diego, CA; synthesized by Vichem Chemie Ltd, Budapest, Hungary) was dissolved in DMSO (Sigma) to form 5 mM solution, and was used in 1 and 5 μM concentrations for *in vitro* studies and 0.5 mg/kg dose was applied for *in vivo* assays. Appropriate concentrations of DMSO solutions were used as control both *in vitro* and *in vivo*.

### Cell Proliferation Assay

HNSCC cells were plated at 2 × 10^3^ viable cells/well on 96-well plates (Greiner, Frickenhausen, Germany) and after attachment to the surface, treated with the EGFR, RAS and c-MET inhibitors at the concentrations described above for 48 h. All treatments were performed in serum-containing or serum-free medium, in a total volume of 200 μl. After the incubation, we added 20 μl of 5 mg/ml thiazolyl blue tetrazolium bromide (MTT, Sigma) to the wells, and incubated for another 4 h at 37°C, then removed the solution, and dissolved the cell culture in 100 μl 1:1 mixture of DMSO and ethyl-alcohol (Sigma). Absorbance was recorded at 570 nm using a microplate Reader (BioRad, Hercules, CA).

### Flow Cytometric Measurement for Apoptosis

PJ15 and PJ41 cell suspensions (1.5 × 105 viable cells/well) were put in 6-well plates, and treated for 48 h with different concentrations of inhibitors: cetuximab (10 or 100 μg/ml), erlotinib (5 or 25 μM), zoledronic acid (10 or 50 μM) and SU11274 (1 or 5 μM). Then, they were detached with 0.02% EDTA solution (Sigma), washed twice with PBS. After centrifugation for 5 min by 1000 rpm, cells were fixed in 1 ml 70% ethanol at –20°C for 30 min and washed in PBS. We added 2 ml Staining Buffer containing 12 μl propidium iodide and 6 μl RNase (Partec, Munster, Germany) to each sample. After 4 h of incubation we quantified the total DNA content in the cells by flow cytometry (Cy-Flow SL-Green, Partec). Apoptotic cells were determined as the amount of cells in the sub-G1 state of propidium iodide (PI) staining. Samples were analyzed by the FlowMax software Sysmex (Partec, Görlitz, Germany).

### Flow Cytometric Measurement of c-MET and EGFR Protein Expression and Activation

Cultured cells were detached using 0.02% EDTA solution, washed two times with serum-free medium, and then fixed/permeabilized in 1% ice-cold methanol for 15 min. Nonspecific binding sites were blocked with 3% BSA for 15 min. After that, cells were labeled with the following antibodies: polyclonal rabbit antibody against the intracellular domain of EGFR (1:10 dilution in PBS, PU335-UP, BioGenex, San Remon, CA); phospho-specific polyclonal rabbit antibody binding to the phospho-tyrosine autophosphorylation site 1068 on the tyrosine kinase domain (1:100 dilution in PBS, 44–788, Biosource International Inc., Camarillo, CA); polyclonal rabbit antibody against the intracellular domain of c-MET (1:50 dilution in PBS, C-12, Santa Cruz Biotechnology, CA) and rabbit anti-c-MET [pYpYpY1230/1234/1235] phosphospecific antibody (1:20 dilution in PBS, Biosource, Nivelles, Belgium) for 45 min at 37°C. After washing thrice for 5 min (PBS), FITC-conjugated polyclonal goat anti-mouse antibody (DakoCytomation, Glostrup, Denmark) was used as secondary antibody. Fluorescence values were detected by FACSCalibur flow cytometer (Becton Dickinson, Sunnyvale, CA). Positive events were counted from at least a total of 104 cells. Negative controls were prepared using isotype control IgG (Sigma).

### Western Blot Analysis

One million adherent cells cultured and drug treated in T25 cell culture flasks were lysed with MLB (No. 20–168, Merck KGaA, Darmstadt, Germany) cell lysis solution containing Halt protease inhibitor cocktail (Fisher Scientific) and 0.1 mg/ml PMSF to inhibit protein and phospho-protein degradation. Following SDS-PAGE and blotting, protein levels were detected using the following primary antibodies: rabbit polyclonal pERK1/2 and ERK1/2 (#9101 and #9102, respectively, Cell Signaling Technology), at a dilution of 1:1,000, phosphospecific polyclonal rabbit antibody against phospho-tyrosine 1,068 (Biosource 44–788) at a dilution of 1:500, and mouse IgG1 antibody against the extracellular domain of EGFR (Clone E30, M7239, Dako). We detected antibody signals using appropriate HRP-conjugated goat secondary antibodies (Jackson ImmunoResearch, West Grove, PA, United States). Immunoblots were revealed by ECL Reagent (GE Healthcare, Dassel, Germany).

### Anti-EGFR Therapy Effects on Subcutaneously Growing Tumor Xenografts

SCID (CB17/Icr-*Prkdc* ^scid^) mice were originated from our inbred and housed SPF (specific pathogen-free) mouse colony. Not more than 10 animals were caged together. Previously cultured and trypsinized PE/CA-PJ15, and PE/CA-PJ41 cell suspensions washed with serum-free medium was inoculated subcutaneously into the back of 8–10-week old treatment-naïve male SCID mice, at a quantity of 1.6 × 10^6^ cells/animal, 7 animals in each groups. Twenty-four days following subcutaneous injection of the PJ15 and thirty-six days following the injection of the PJ41 tumor cells, measurable tumors were developed, the animals were assigned to treatment groups in order to have the same tumor size distribution at the beginning of the treatment. To record the effects of targeted therapies on tumor growth, animals were treated intraperitoneally (i.p.) with the following inhibitors for three or four weeks (PJ15 and PJ41, respectively): erlotinib (2 mg/kg, daily), cetuximab (400 + 250 mg/m^2^, weekly), zoledronic acid (50 μg/kg, weekly), erlotinib + cetuximab, erlotinib + zoledronic acid, cetuximab + zoledronic acid, erlotinib + cetuximab + zoledronic acid and physiological saline solution as negative control.

The volumes of the subcutaneously growing tumors were determined by the measurement of two diameters by caliper twice weekly from the first treatment. Tumor volumes were estimated using the formula [length × width^2^ × π/6]. The treated groups were compared to untreated group; the relative effects of the treatments were analyzed using percentages of the control tumor growth.

### Murine Liver Colonization Assay for Human Tumor Xenografts

Previously cultured human HNSCC PE/CA-PJ15 and PJ41 cells were trypsinized, washed twice with serum-free medium, and suspension containing 5 × 10^5^ cells was inoculated into the spleen of anesthetized 8–10-week old treatment-naïve male SCID mice (8 animals in each group). Twenty-eight days after tumor cell injection, intraperitoneal administration of 0.5 mg/kg SU11274 or solvent control occurred daily for 3 weeks. At the endof the experiment, tumors were excised, tumor mass was measured, and macroscopic liver colonies were counted using a stereomicroscope. The effect of SU11274-treatment was determined based on tumor weight or metastasis number as a percentage of those of the control group. Tumor cells were labeled with anti-cytokeratin antibody (AE1/AE3, M3515, Dako) according to manufacturer’s recommendations using AEC as chromogen.

### Animal Housing and Treatment Conditions

All animal housing and breeding processes (permission No: PEI/001/1738-3/2015) and animal experiments (permission No: PEI/001/2574–6/2015) were conducted following standards and procedures approved by the Animal Care and Use Committee of the National Institute of Oncology, Budapest. Group sizes were reduced to minimize animal use, while representing well the individual variances inside the group. All (112/112) animals inoculated subcutaneously were included in the analysis. In the liver metastasis model, we excluded five animals where no visible sign of primary or metastatic tumor burden occurred, signaling inefficient intrasplenic injection. In total, 27/32 animals were included in the metastasis analysis. All inoculations and treatments were performed under a sterile laminar biosafety cabinet (between 1:00 PM and 3:00 PM each treatment day). Tumor cell inoculation was performed always on animals anesthetized by the intraperitoneal injection of the mixture of zolazepam (20 mg/kg), xylazine (12.5 mg/kg), butorphanol (3 mg/kg), and tiletamine (20 mg/kg). Assessment of proper anesthetization was controlled by pedal reflex and monitoring respiratory rate. All animals were euthanized using anesthetization by isoflurane inhalation followed by cervical dislocation. Our animal house follows FELASA health status control recommendation to monitor whether all our strains are free from pathogens or other diseases. All animals recruited to the experimental group weighed between 22 and 25 g at the beginning of the experiment, and no more than 10% body weight loss was observed through the experiments.

### Statistics

To determine statistical differences between two groups Student’s t-test was used. For statistical analysis of differences among more than two groups ANOVA was used with the *post hoc* Scheffé-test, where parametric methods were available. For the animal experiments we used the non-parametric Mann-Whitney *U*-test to evaluate treatment effects. Statistical significance was determined and marked as following: *: *p* < 0.05; **: *p* < 0.01; ***: *p* < 0.001. Statistical analysis was realized using Statistica 12.0 software (StatSoft, Tulsa, OK).

## Results

### Effect of the EGFR Polymorphism R521K on the *In Vitro* Sensitivity of HNSCC Cell Lines to the EGFR Specific Therapeutic Antibody Cetuximab, and Small-Molecule Inhibitors of EGFR, RAS, and c-MET

**FIGURE 1:**
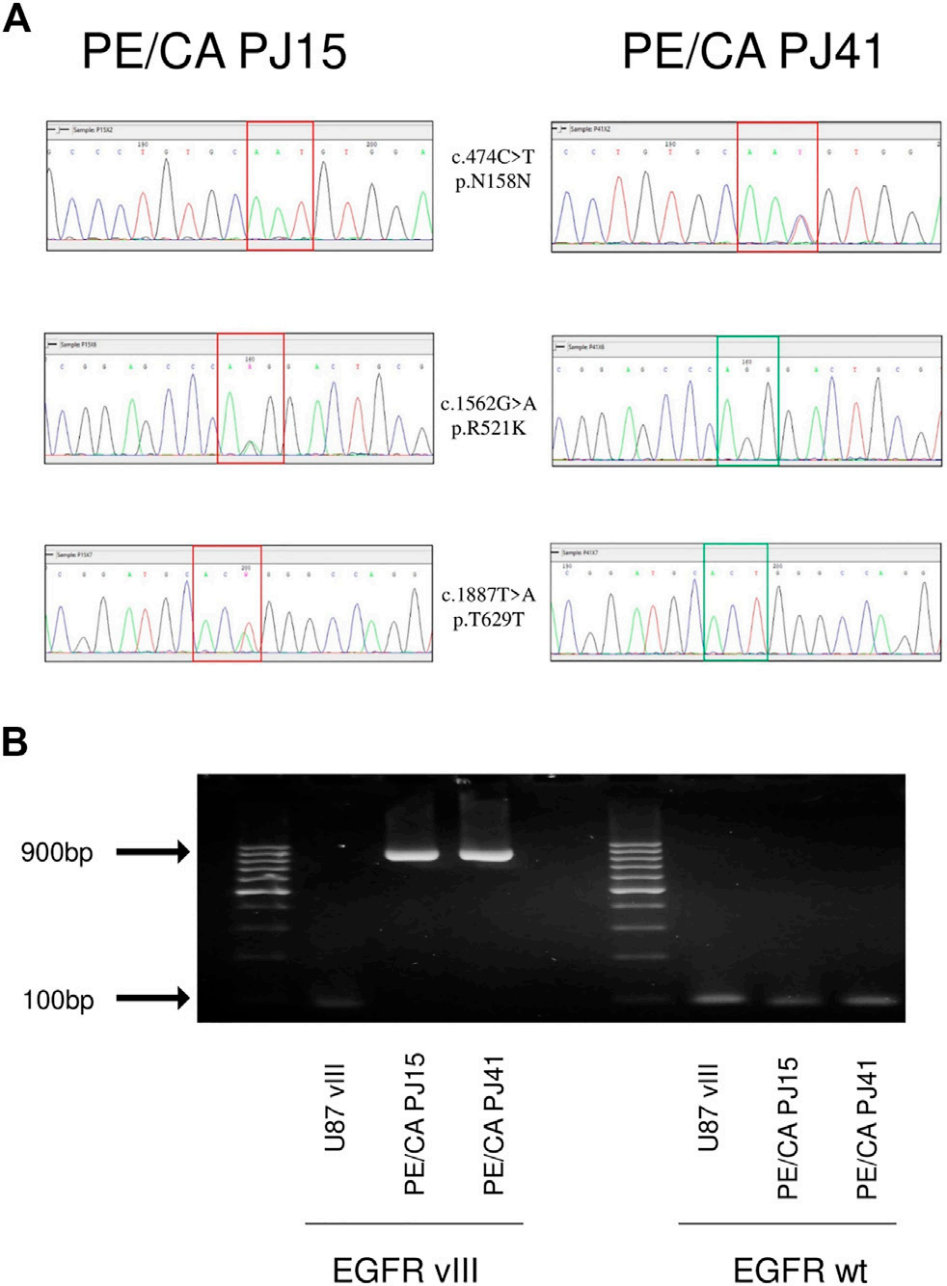
Genetic alterations of EGFR extracellular domain in PE/CA-PJ15 and PJ41 HNSCC cell lines **(A)** Different SNP pattern in the case of two PE/CA cell lines: PE/CA-PJ15 c720 C > T Asn158Asn homozygous SNP **(upper row, left)**, PE/CA-PJ41 c720 C > T Asn158Asn heterozygous SNP **(upper row, right)**, PE/CA-PJ15 c1808 G > A, Arg521Lys **(middle row left)**; PE/CA-PJ15 c2133 T > A Thr629Thr **(lower row, left)**. **(B)** investigation of EGFR vIII and EGFR wild type isoforms after mRNA isolation, reverse transcription, PCR and agarose gel electrophoresis. The left side shows that U87 vIII, an EGFR vIII overexpressing glioblastoma cell line expresses the vIII isoform of the receptor, while the two HNSCC cell lines express only the untruncated wild type isoform, showing the amplification of a large region of 896 base pairs. The right side of the image shows that wild type EGFR is present in all of the samples.

To investigate the possible effects of EGFR genetic alterations on the sensitivity of HNSCC cell lines to the EGFR-directed antibody cetuximab, the EGFR specific tyrosine kinase inhibitor (TKI) erlotinib, the RAS inhibitor zoledronic acid, and the c-MET inhibitor SU11274, we chose two cell lines, the PE/CA-PJ15 harboring one R521K mutant allele of EGFR (heterozygous) and the PE/CA-PJ41 cell line expressing only wild type EGFR (homozygous wild type, Figure 1A). Molecular biological analysis of the extracellular domain also revealed two other synonym SNPs in the EGFR gene (Figure 1A). The two cell lines were found to be negative for the EGFRvIII isoform (deletion of 2-7 exons, results shown on Figure 1B) and for the mutations of the TK domain (regions exon 19 and exon 21), and did not contain any alterations in the hot point regions of KRAS and NRAS, either (see Supplementary Table S2).

**FIGURE 2:**
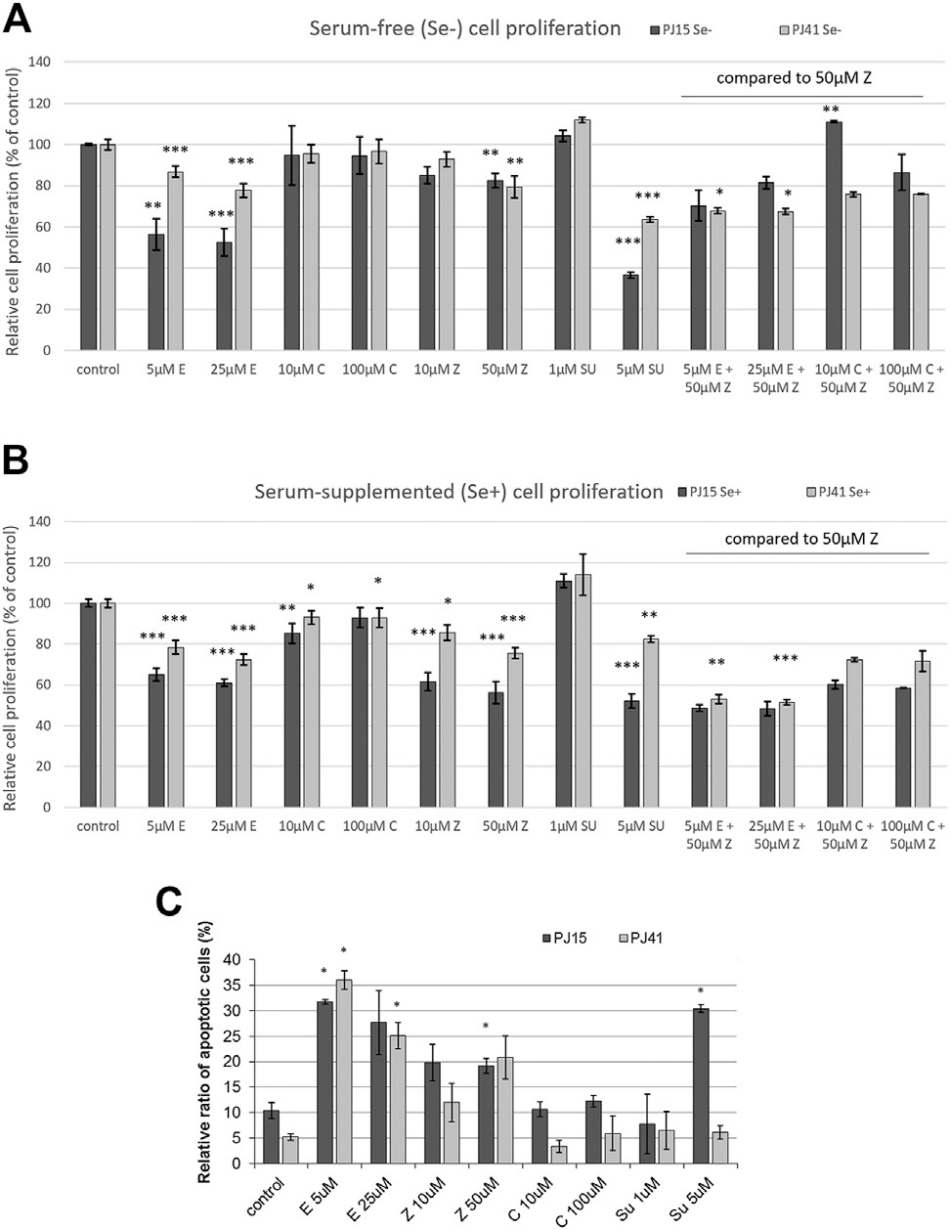
Effects of investigated inhibitors on the *in vitro* proliferation and apoptosis of HNSCC cell lines. **(A,B)** While therapeutic antibody cetuximab had no effect on *in vitro* cell proliferation in any of the two cell lines, small molecule EGFR- and c-MET-specific TKIs and RAS inhibitors were found to be more effective against PJ15 cells compared to PJ41. The combined therapy of HNSCC cells with anti-EGFR compounds shows that cetuximab did not affect cell proliferation, not even in combination, but TKIs and RAS inhibitor have pronounced antiproliferative effect on the two cell lines, which was much stronger on PJ15 cells than on PJ41 cells. Graphs show mean ± SD of four parallel samples, for single drug treatments, and two parallels of combination treatments, quantified as % of control (untreated cells). All values were measured in triplicates. **(C)** Flow cytometric determination of apoptotic nuclei (subG1 fraction). Erlotinib and zoledronic acid significantly increased the apoptosis in both HNSCC cell lines. However, c-MET-specific inhibitor SU11274 induced apoptosis in the PJ15 cells only. Cetuximab had no effect similarly to results of the proliferation assay. Data are means ± SD of three parallel samples “C”: cetuximab; “E”: erlotinib, “Z”: zoledronic acid; “SU”: SU11274.

To evaluate the sensitivity of HNSCC cell lines, first we investigated the *in vitro* effects of EGFR, RAS and c-MET inhibitors on cancer cell proliferation. While the EGFR specific TK inhibitor erlotinib and the RAS inhibitor zoledronic acid significantly decreased *in vitro* proliferation of the two HNSCC cell lines both in serum-containing and serum-free media (Figures 2A,B), interestingly, the therapeutic anti-EGFR antibody cetuximab showed no effect on cancer cell proliferation either in the case of the PE/CA-PJ15 cell line harboring the R521K EGFR polymorphism or in the homozygous wild type PE/CA-PJ41 cells. Moreover, we found that the specific c-MET inhibitor SU11274 at higher concentration efficiently decreased cancer cell proliferation in both PE/CA-PJ41 and PJ15 cells. However, the sensitivity of the two cell lines against the specific small-molecule inhibitors was different as shown in Figures 2A,B. PE/CA-PJ15 cell line had higher sensitivity in all cases, especially when cells were treated with the c-MET inhibitor SU11274. To further investigate the similar cetuximab resistant behavior of the two cell lines, we also measured the combined effects of the inhibitors. We observed that the toxicity of cetuximab was not augmented by the presence of the RAS inhibitor zoledronic acid even at high antibody concentration (100 μg/ml) verifying the *in vitro* cetuximab resistant phenotype of PE/CA-PJ15 and PJ41 cells. Contrarily, the combination of erlotinib and zoledronic acid showed clear additive cytotoxic effects in PE/CA-PJ41 cells. Next, we monitored drug/antibody induced apoptosis in HNSCC cell lines on the basis of the decreased DNA content of apoptotic cells (sub-G1 population) by flow cytometric analysis. Consistent with our previous results, cetuximab treatment did not induce apoptosis in any of the two cell lines confirming *in vitro* ineffectiveness of cetuximab (Figure 2C). Contrarily, small-molecule inhibitors significantly increased apoptotic cell number in both cell lines. Moreover, the c-MET inhibitor SU11274 provoked dramatic cell death in PE/CA-PJ15 cells, while in PE/CA-PJ41 cells the apoptotic cell number did not increase above the baseline (untreated control).

### *In Vivo* Cetuximab Resistance of PE/CA-PJ15 Cells Harboring the R521K EGFR Polymorphism

**FIGURE 3:**
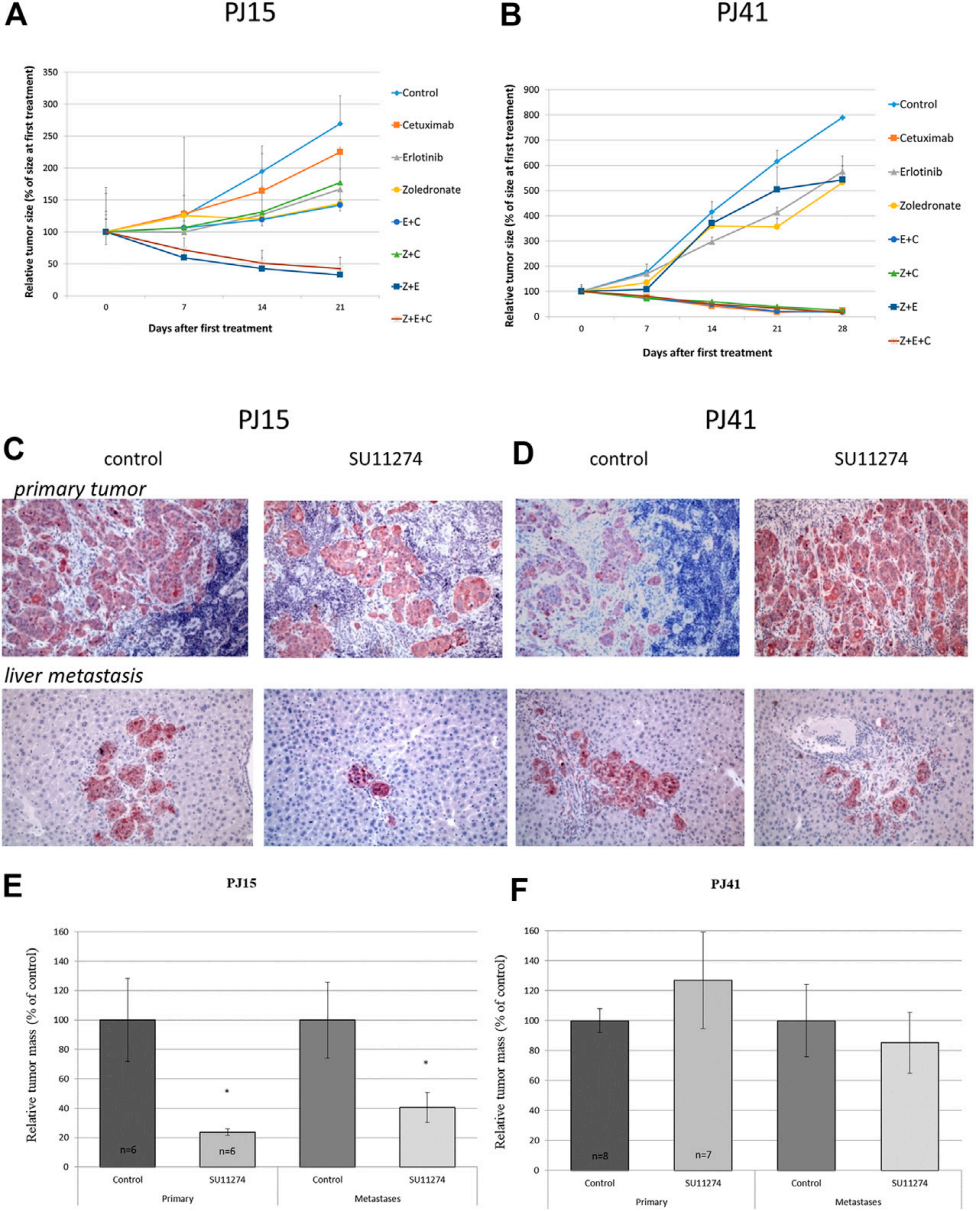
*In vivo* effect of EGFR-, RAS- and c-MET-inhibitors on growth and metastatic colonization of HNSCC xenografts Subcutaneously growing HNSCC tumor xenografts (**(A)** PE/CA-PJ15, **(B)** PE/CA-PJ41) bearing mice were treated i.p. with the EGFR, Ras and c-MET inhibitors mono- and combination therapy as well. In the case of PJ15 cetuximab had no effect on tumor growth, but erlotinib and zoledronic acid decreased significantly the tumor volume either in monotherapy or in combination treatment. However, cetuximab had profound effect on tumor growth of PJ41 xenograft: all the investigated cetuximab-containing therapeutic regimes decreased dramatically the tumor volume. **(C–D)** Immunohistochemical detection of tumor cells using cytokeratin antibody in spleen **(*upper row*)** and liver **(*lower row*)** after colonization assay. In the case of cetuximab-resistant PJ15 **(C)** SU11274 treatment (*right pictures in the panel*) significantly decreased both the primary tumor mass and the number of tumor colonies in the liver compared to control (*left pictures*). The primary tumor mass was decreased to 20% of the control in treated animals, and the number of the liver colonies were decreased to less than 40% of the control group **(E)**. However, SU11274 had no effect on either the primary tumor growth or liver colonization of cetuximab-sensitive PJ41 xenografts **(F)**. Graph represent means ± SEM. Significance was measured by Mann-Whitney *U*-test. “C”: cetuximab; “E”: erlotinib, “Z”: zoledronic acid; “SU”: SU11274.

To characterize the *in vivo* sensitivity of HNSCC cells against the specific inhibitors, SCID mice bearing PE/CA-PJ15-, and PE/CA-PJ41-derived xenografts were treated with the inhibitors intraperitoneally in human equivalent doses. Interestingly, in contrast to our *in vitro* results, the two HNSCC cell lines showed markedly different sensitivity to cetuximab. In the case of PE/CA-PJ15 cells harboring the R521K EGFR mutation, cetuximab did not affect tumor growth. However, the treatment with erlotinib or zoledronic acid significantly decreased PJ15 tumor volumes (Figure 3A). Moreover, combined treatments acted additively in all therapeutic regimens confirming the ineffectiveness of cetuximab and revealing the most efficient drug combination of erlotinib and the RAS inhibitor zoledronic acid that dramatically eliminated cetuximab resistant tumors (Figure 3A). Contrarily, cetuximab showed profound inhibitory effect on the growth rate of PE/CA-PJ41-derived xenografts expressing only wild type EGFR, and tumors were completely eradicated upon treatment with all combined therapeutic regimes containing cetuximab (Figure 3B). Interestingly, in the case of PE/CA-PJ41-derived tumors, the combination of erlotinib and zoledronic acid did not enhance treatment efficiency but showed the same inhibitory effect as the drugs alone.

### *In Vivo* Cetuximab Resistance Is Accompanied by a Profound *In Vivo* Sensitivity to the c-MET Specific Tyrosine Kinase Inhibitor SU11274

Given that c-MET and EGFR signaling pathways share molecular nodes (PI3K/Akt, MAPK), and recent data show the possible compensatory function of these receptor tyrosine kinase stimulated pathways [25], we examined the *in vivo* effect of the c-MET inhibitor SU11274 on primary tumor growth and liver colonization ability of the cetuximab resistant PE/CA-PJ15 and the cetuximab sensitive PJ41 HNSCC cells. Twenty-eight days after intrasplenic inoculation of tumor cells SCID mice were treated with SU11274 daily for 4 weeks. The effect of SU11274 treatment was monitored immunohistochemically using the epithelial cell-specific human cytokeratin antibody. SU11274 treatment significantly decreased primary tumor mass and even more dramatically reduced the number of hepatic tumor colonies in the case of the cetuximab resistant PE/CA-PJ15 cells (Figures 3A,C,E). However, the c-MET inhibitor did not have any significant effect either on primary tumor growth, or on liver colonization ability of the cetuximab sensitive PE/CA-PJ41 cancer cells (Figures 3B,D,F).

### Expression and Activation Status of EGFR and c-MET in PE/CA-PJ15 and PE/CA-PJ41 Cells

**FIGURE 4:**
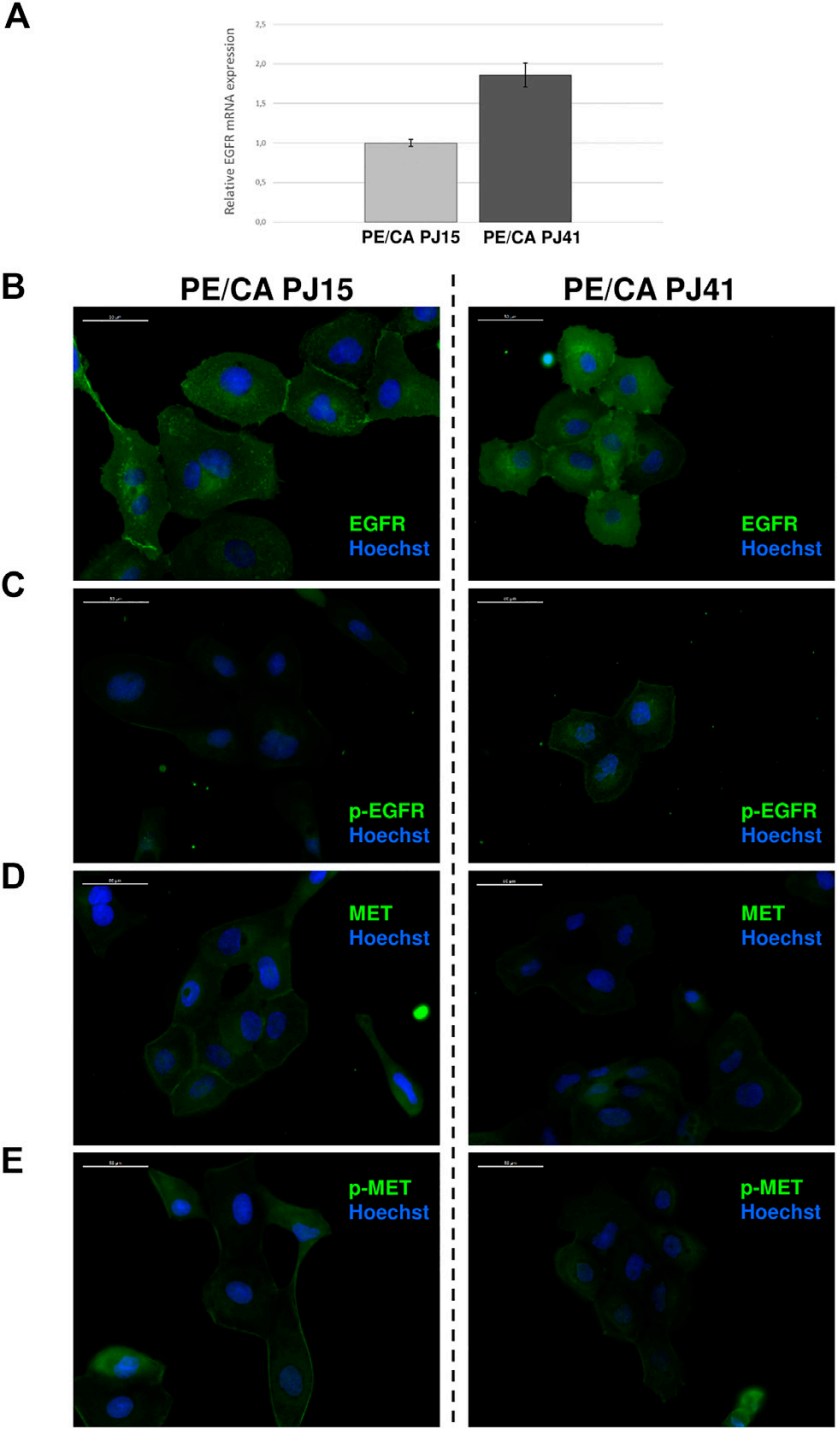
Expression of EGFR and c-MET in HNSCC cell lines. EGFR mRNA expression measured by quantitative real-time PCR was nearly 2-fold higher in PE/CA-PJ41 cells compared to the PJ15 cell line **(A)**. Immunocytochemistry of PJ15 **(*left*)** and PJ41 **(*right*)** showed that both HNSCC cell lines express the EGFR **(B)** and c-MET **(D)** proteins. Moreover, these receptors are active without exogenous ligand activation in these cells detected by phospho-specific antibodies recognizing the active receptors **(C,E)**. However, while EGFR (in line with the flow cytometric data in Table 2) expression is higher in PJ41 cells **(B)**, c-MET and p-c-MET expression was slightly higher in PJ15 cells **(D,E)**. White bars mark 50 μm distance.

Since the amount of plasma membrane-localized EGFR fundamentally influences the effectiveness of erlotinib and cetuximab treatments, we investigated receptor expression first at the DNA level. Based on FISH results, we found that both cell lines expressed normal EGFR gene copy number as we could not detect either gene amplification, or polysomy (see Supplementary Table S2). Next, we measured EGFR mRNA expression by quantitative real-time PCR detecting a 2-fold increase in receptor expression in PE/CA-PJ41 cells compared to the PJ15 cell line (Figure 4A). To check whether this difference is also manifested at the protein level, the ratio of EGFR positive cells and the level of expression were analyzed by flow cytometry and immunocytochemistry. We detected similar protein expression in PE/CA-PJ15 and PJ41 cells stained with an intracellular domain-specific EGFR antibody. However, the fluorescence intensity was weaker in PJ15 cells harboring the R521K EGFR mutation compared to PJ41 in the case of the extracellular domain-specific labeling (Table 2; Figure 4B). Moreover, we observed that 90% of the cells were positive for phospho-EGFR (Tyr^1068^) in both cell lines and even the expression level was found to be similar (Table 2; Figure 4C). These results raised the possibility that not the amount of the receptor is responsible, but the binding efficiency of the extracellular epitope-specific antibody differs between PE/CA-PJ15 and PJ41 cells. c-MET expression was also evaluated by the above methods, and we found that the percentage of the positive cells, expression level and activity were significantly higher in case of PE/CA-PJ15 cells compared to PJ41 (Table 2; Figures 4D,E).

**TABLE 2:** Labeling of HNSCC cells with EGFR and c-MET antibodies using flow cytometry.

	PE/CA-PJ15		PE/CA-PJ41	
	% of positive cells	Mean intensity	% of positive cells	Mean intensity
Control	4.1 ± 1.13	57.0 ± 1.41	0.95 ± 0.07	44.0 ± 0.01
EGFR extracellular antibody	75.0 ± 4.24	74.3 ± 0.71	91.5 ± 2.12	107.0 ± 1.42
EGFR intracellular antibody	98.4 ± 0.71	122.5 ± 3.54	91.75 ± 1.77	110.0 ± 4.24
p-EGFR	90.4 ± 2.26	97.0 ± 1.41	89.0 ± 1.41	76.5 ± 3.46
c-MET	95.4 ± 1.34	110.5 ± 3.55	79.5 ± 6.36	97.25 ± 3.89
p-c-MET	94.8 ± 0.57	102.0 ± 1.45	78.0 ± 2.83	89.8 ± 1.27

Proportion of positively gated cells and mean fluorescence intensity values show similar labeling in PE/CA-PJ15 and PE/CA-PJ41 cells. All data represent mean ± SD values from 3 parallel measurements.

### Effect of EGFR, RAS and c-MET Inhibition on EGFR and ERK Activation in PE/CA-PJ15 and PE/CA-PJ41 Cells

**FIGURE 5:**
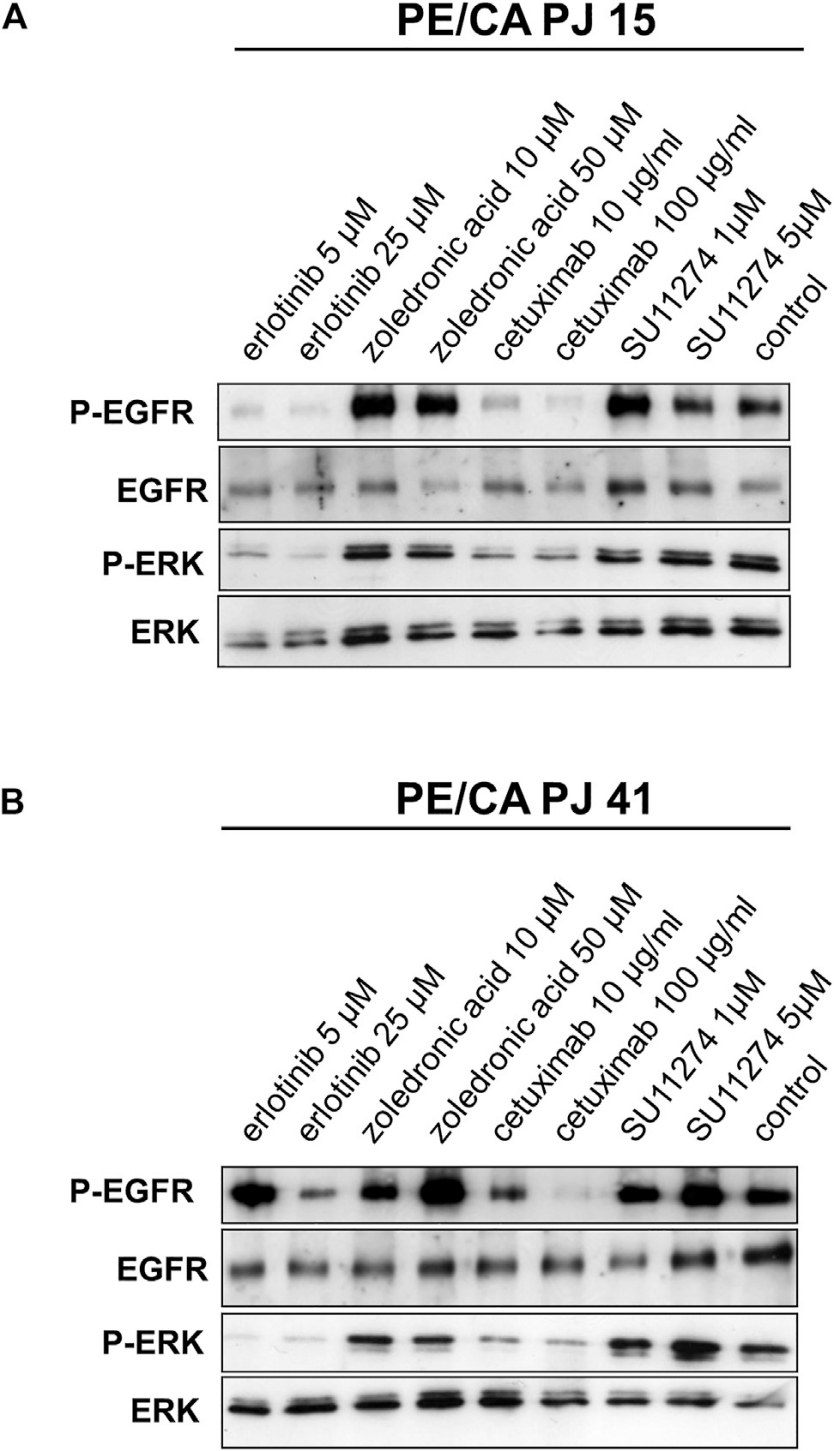
Effect of EGFR, RAS and c-MET inhibition on EGFR and ERK activity in PE/CA-PJ15 and PE/CA-PJ41 cells. Cetuximab treatment effectively decreased EGFR and ERK activity in both cell lines. The signaling activity was measured by Western blot using p-EGFR and p-ERK antibodies. The level of inhibition was comparable to the effect of erlotinib treatment and was more pronounced in PE/CA-PJ15 cells. The total EGFR and ERK labeling served as loading control.

To investigate whether the suggested decreased efficiency of cetuximab binding to the R521K mutant extracellular domain of EGFR in PE/CA-PJ15 cells results in a less effective inhibition of EGFR phosphorylation, we monitored the level of phospho-EGFR and phospho-ERK in HNSCC cells by immunoblots. Interestingly, we found that cetuximab treatment effectively decreased EGFR and ERK activity in both cell lines even at low concentration of 10 µM. Moreover, the inhibition effect was comparable to that of erlotinib treatment, and was more pronounced in PE/CA-PJ15 cells (Figure 5). These findings suggested that the *in vivo* sensitivity of HNSCC cells to cetuximab is not strictly related to the disruption of the tumor cell proliferation-supporting activity of the EGFR/ERK pathway. Our results raise the possible role of other RTK-stimulated pathways, and the involvement of the anti-EGFR antibody induced cytotoxic immune response in tumor elimination.

## Discussion

EGFR overexpression and mutation have been described as an oncogenic driver in several tumor types including HNSCC, making it a promising target for anticancer therapies in the last decade. Clinical trials showed that in patients with locally advanced HNSCC, cetuximab in combination with radiotherapy significantly increases the progression-free and overall survival of the patients [10]. Another clinical trial compared platinum-based plus fluorouracil combination chemotherapy with cetuximab or alone among patients with recurrent or metastatic squamous-cell carcinoma of the head and neck region. The result was similar: overall and progression-free survival were improved with cetuximab-combined concomitant chemotherapy [11].

However, additional targeted therapy improves clinical outcome only in a limited part of the patients, and lack of durable effect limits the clinical benefit of cetuximab in HNSCC patients [26].

Several studies have examined the possible causes of the primary and acquired resistance of EGFR blockade, and their findings suggest that not only the EGFR gene alterations alone, but many other signaling pathways play important role in the resistance to anti-EGFR therapy [27].

In our study, we examined two HNSCC cell lines to investigate possible causes of the failure of targeted therapy. Molecular biological analysis of EGFR ECD showed that four SNPs were found in the HNSCC cell lines. One of them is a guanine to adenine mutation, which causes an arginine to lysine amino acid change at codon 521 (c1808, Arg521Lys), leading to the EGFR R521K heterozygous polymorphism in case of PJ15 cells.

During *in vitro* proliferation assays, we measured significant inhibition on both PE/CA-PJ41 and PJ15 cells using the EGFR specific TK inhibitor erlotinib and the RAS inhibitor zoledronate, but interestingly, cetuximab, already used in clinical practice, was ineffective, suggesting that the mechanism of action underlying cetuximab therapy might be different than direct cell proliferation inhibition, or apoptosis induction.

In the combination treatment experiments, erlotinib and zoledronate in combination had the most potent antiproliferative effect, but the additional combination with cetuximab could not suppress cell proliferation in either cell lines any further.

We could further demonstrate the *in vitro* ineffectiveness of cetuximab: in the drug/antibody induced apoptosis assay cetuximab treatment did not induce apoptosis either in PJ15, or PJ41 cell cultures. Also, in line with the results of the *in vitro* proliferation assays, the treatment with TK inhibitor erlotinib and RAS inhibitor zoledronate caused significant cell death in both cell lines. Furthermore, we observed that the c-MET inhibitor SU11274 provoked remarkable apoptosis in PJ15 cells (harboring the R521K EGFR polymorphism), while it was ineffective in the homozygous wild type PE/CA-PJ41 cells.

Cetuximab is a monoclonal antibody and has a dual mechanism of action *in vivo*. Primarily, it binds the extracellular ligand-binding domain of the EGF receptor, thereby blocking the transmission of information through the specific signaling pathway and evokes a cascade of complex antitumor effects, including cell-cycle arrest, induction of cell death, inhibition of angiogenesis and metastasis, and downregulation of EGF receptor expression [28]. Besides, there are other publications assuming that cetuximab binding induces various immune interactions via its Fc fragment. The tumor cells with bound cetuximab antibodies are more easily detected by NK cells, and other nonspecific cytotoxic cells which leads to antibody-dependent cellular cytotoxicity (ADCC). In addition, binding the components of complement cascade through the Fc region, cetuximab may initiate complement-dependent cytotoxicity (CDC) as well [29,30].

In our *in vivo* experiments, SCID mice bearing PE/CA-PJ15-and PJ41-derived xenografts were treated with specific targeted inhibitors, such as erlotinib, zoledronate and cetuximab. Unlike in our *in vitro* assay models, the two HNSCC cell lines showed significantly different sensitivity to cetuximab *in vivo*. In case of PE/CA-PJ41 cells harboring the wild type EGFR, cetuximab showed dramatic inhibitory effect on the tumor growth: tumors were completely eradicated upon treatment with all combined therapeutic combination containing the monoclonal antibody. Nevertheless, PE/CA-PJ15 cells, expressing the R521K mutant variant of the EGF receptor, showed no response to cetuximab treatment, in line with our *in vitro* findings. Cetuximab barely affected tumor growth, while the co-treatment with erlotinib and zoledronate decreased tumor proliferation significantly. The effect of erlotinib and zoledronate in combination could not be achieved by any other therapeutic regimens.

Similar observations were made in flow cytometric and immunohistochemical experiments, since we detected nearly the same EGFR protein expression in PE/CA-PJ15 and PJ41 cells with the IC domain specific antibody, but the fluorescent intensity with the EC domain specific antibody was significantly weaker in the mutant cell line than in PJ41. Moreover, we found that cetuximab treatment effectively reduced the level of phospho-EGFR and phospho-ERK in both cell lines on immunoblots. This also means that, despite the decreased efficiency of cetuximab binding, PE/CA-PJ15 cells with the R521K mutant receptor still undergo the inactivation of EGFR activity.

These results raised the possibility that EGFR R521K polymorphism acts for an important mechanism of intrinsic resistance to cetuximab therapy, since it causes decreased binding efficiency of the extracellular epitope specific antibody. These findings also suggest the involvement of the anti-EGFR antibody induced cytotoxic immune response in tumor elimination and that multi-targeting approach to the EGFR signaling cascade may eliminate cetuximab resistant tumors.

Recently, numerous studies have indicated that activation of alternative pathways and receptors might be the reason of the acquired failure of anti-EGFR therapy. Many pre-clinical studies have described that HER family receptors are compensatory overexpressed when EGFR is blocked, leading to therapy resistance [17]. Another study demonstrated that HER3 receptor is upregulated and activated in HNSCC cells after cetuximab therapy, which negatively influences the response to mAb treatment [31]. Activation of HER2 and HER4 receptors has been also reported as a potential key player in the mechanism of cetuximab resistance. Their signaling pathways share downstream effectors with EGFR, by which HNSCC cells could escape form cetuximab inhibition [17,32]. Due to extensive crosstalk among HER receptors, targeting multiple members of the HER receptor family with pan-ErbB inhibitors (dacomitinib, afatinib) could reach explicit therapeutic benefit and overcome resistance to EGFR targeted therapies [32,33].

Besides HER family and its downstream signaling proteins, in the presence of EGFR blockade, activation of several alternative growth factor receptor pathways has been reported in recent years, such as ALK, VEGF, IGF-1, MET [25,34,35]. In our extended experiments with the specific c-MET inhibitor SU11274, we found that it effectively decreases *in vitro* proliferative capacity of both cell lines. Moreover, we observed that this TK inhibitor provoked remarkable apoptosis in PJ15 cells harboring the R521K EGFR polymorphism, while it was ineffective in the homozygous wild type PE/CA-PJ41 cells.

Based on our immunofluorescent and flow cytometric assays, we can assess that the c-MET receptor was expressed by both cell lines and was active without the presence of its extracellular ligand, but the phosphorylated c-MET signal in the PJ15 cell line was significantly more active compared to the other cell line. This result coincides with the observation [17] that c-MET receptor is present in constitutively activated form in cetuximab resistant cells.

Since aberrant c-MET function plays an important role in the metastatic capacity, we examined the *in vivo* effect of the c-MET inhibitor SU11274 on primary tumor proliferation and liver colonization ability of the cetuximab resistant PE/CA-PJ15 and the cetuximab sensitive PJ41 HNSCC cells. In the PE/CA-PJ15 cell line the c-MET inhibitor significantly reduced primary tumor growth and even more decreased hepatic colonization. In contrast, in the cetuximab sensitive PE/CA-PJ41 cell line, we could not observe significant effect either on the size of primary tumor mass, or on liver colonization ability.

Taken together, the HNSCC cell line expressing the R521K mutant form of the EGF receptor did not respond well to cetuximab treatment. Prior to initiating antibody therapy in the clinical practice, it would be beneficial to detect the presence of the R521K polymorphism, as it may allow to identify a substantial group of the patients with intrinsic resistance to the monoclonal antibody. However, this connection should be further investigated to see whether R521K polymorphism leads to cetuximab resistance. A recent study found that both *in vivo* xenografts and clinical samples follow the pattern suggested [18].

Based on our results, tumors that are only moderately sensitive to anti-EGFR antibody therapy can respond well to c-MET receptor targeting, but in the case of wild-type EGFR-expressing HNSCCs, there is a major role in signal transmission through the EGF receptor, the anti-EGFR antibody therapy seems mostly effective.

New therapeutic strategies such as inhibitory combinations or the use of multi-targeting inhibitors may provide a breakthrough in the treatment of cetuximab-resistant tumors. Also, combination therapy of EGFR inhibitors and c-MET inhibitors is a promising way to enhance *in vivo* and clinical response to targeted therapy in advanced HNSCC, rising new possibility to patients in need of an efficient therapy.

## Conclusions

As EGFR-targeting treatments in advanced HNSCC patients often fail to express antitumor activity, there is an urge to select patients eligible for anti-EGFR therapy and create novel modalities for those who are not. Based on our *in vitro* and *in vivo* results, EGFR R521K polymorphism can be a potential predictive marker for cetuximab therapy response. Importantly, c-MET-targeted therapy might be the key to successfully achieve clinical response of the tumors showing resistance to EGFR-targeted therapies.

## Data Availability

The raw data supporting the conclusion of this article will be made available by the authors, without undue reservation.

## Abbreviations

AEC, 3-amino-9-ethylcarbazole; ATCC, american type culture collection; c-MET, mesenchymal epithelial transition factor; dNTP, deoxy-nucleotide-triphosphate; ECACC, European collection of cell cultures; EGFR, epidermal growth factor receptor; FDA, food and drug administration; FITC, fluorescein-5-isothiocyanate; HNSCC, head and neck squamous cell carcinoma; IC domain/EC domain, intracellular domain/extracellular domain; KRAS, Kirsten rat sarcoma 2 viral oncogene homolog; NSCLC, non-small-cell lung carcinoma; PCR, polymerase chain reaction; RTK, receptor tyrosine kinase; SNP, single nucleotide polymorphisms; TKI, tyrosine kinase inhibitor
