# Supplementary material for: EGFR Alterations Influence the Cetuximab Treatment Response and c-MET Tyrosine-Kinase Inhibitor Sensitivity in Experimental Head and Neck Squamous Cell Carcinomas
Source: Pathol Oncol Res. 2021 May 3;27:620256. doi: 10.3389/pore.2021.620256 (PMC8262169; doi:10.3389/pore.2021.620256)
Supplement: Supplementary file 1 [file DataSheet1.docx]

**SUPPLEMENTARY MATERIAL**

**EGFR alterations influence the cetuximab treatment response and c-MET tyrosine-kinase inhibitor sensitivity in experimental head and neck squamous cell carcinomas**

Györgyi A. Nelhűbel^1,2^, Mihály Cserepes^1^, Balázs Szabó^3^, Dóra Türk^1^, Adél Kárpáti^1^, István Kenessey^4,5^, Erzsébet Rásó^4^, Tamás Barbai^4^, Zita Hegedűs^1^, Viktória László^6,7^, Bálint Szokol^8^, Judit Dobos^8^, László Őrfi^8^, József Tóvári^1^

^1^ Department of Experimental Pharmacology, National Institute of Oncology, Budapest, Hungary

^2^ 2^nd^ Department of Pediatrics - Semmelweis University, Budapest, Hungary.

^3^ Department of Otolaryngology and Head and Neck Surgery, Semmelweis University, Budapest, Hungary

^4^ 2^nd^ Institute of Pathology, Semmelweis University, Budapest, Hungary

^5^ Hungarian Cancer Registry, National Institute of Oncology.

^6^ Department of Thoracic Surgery, Medical University of Vienna, Vienna, Austria

^7^ Department of Tumor Biology, National Korányi Institute of Pulmonology, Budapest, Hungary

^8^ Vichem Chemie Ltd., Budapest, Hungary

***Correspondence:***

**József Tóvári, PhD.**

Department of Experimental Pharmacology, National Institute of Oncology, Budapest, Hungary

Phone: +36-1-224-8778

Fax: +36-1-224-8724

E-mail: [tozsi@oncol.hu](mailto:tozsi@oncol.hu), [jtovari@yahoo.com](mailto:jtovari@yahoo.com)

**SUPPLEMENTARY MATERIALS AND METHODS**

**Detection of EGFR gene amplification by FISH**

Five-μm-thick sections were cut from paraffin embedded tumors followed by cooking at 56°C overnight. The slides were deparaffinized by soaking in xylene for 10 minutes two times, and rehydrated in a series of decreasing concentrations of ethanol. Heat pretreatment was carried out by microwaving the slides in Vector Antigen Unmasking Solution (H-3300; Vector Laboratories, Burlingame, CA, USA) at 800 W for 3 minutes and at 160 W for further 20 minutes. The tissue sections were then digested with 0.025% pepsin (P7012; Sigma-Aldrich, Saint Luis, MO, USA) in 0.2 M HCl at 37°C for 15 minutes. Pepsin digestion was terminated by washing the slides in distilled water for 1 minute and in 2× SSC for 5 minutes followed by dehydration in a series of increasing concentrations of ethanol. 10 μl of ON EGFR, Her-1 (7p11) / SE 7, dual-color FISH probe mix (KBI-10702, Kreatech Biotechnology B.V., Amsterdam, Netherlands) was applied onto the selected area on each slide and covered with a glass coverslip which was sealed with rubber cement. The slides were incubated at 80°C for 5 minutes for codenaturation of sample and probe DNA, and hybridization was carried out by overnight incubation at 37°C. Posthybridization washes were performed in 0.4× SSC / 0.3% Igepal for 2 minutes at room temperature and in 0.4× SSC / 0.3% Igepal for 2 minutes at 70°C. After dehydration in a series of ethanol, Vectashield mounting medium with DAPI (4'6-diamidino-2-phenylindole, 1.5 μg/mL, Vector Laboratories, Burlingame, CA, USA) was applied for fluorescent nuclear counterstaining. Leica DM RXA motorized epifluorescence microscope (Leica Microsystems, Wetzlar, Germany), equipped with Pieper FK-7512-IQ high sensitivity B&W video camera and Leica CW4000 FISH visualizing software, was used for the analysis and documentation of the FISH results. EGFR gene copy and the chromosome 7 number were counted in 40 cells from the two samples.

**Detection of EGFR TK domain mutations**

We examined the samples’ EGFR mutation status using the method set previously with EGFR wild type H358 bronchioalveolar carcinoma, the 19del mutant HCC827 epithelial adenocarcinoma and the H1975 human pulmonary adenocarcinoma with a point mutation in exon 21 cell lines as positive controls. The control samples contained WT DNA only, or WT and mutant DNA in 5%, 17.5%, and 50% ratio. After normalization we were able to differentiate samples containing WT from low concentration mutant alleles. During the experiment we used the Roche LightCycler^®^ 480 Real Time PCR System, adding to the 20 µl total volume reaction mixture 10µl High Resolution Melting Master (Roche), 0.5-0.5 µl of 10 µM forward and reverse primers, 2.5 µl 25 mM MgCl_2_ and 25 ng DNA template. We used the following primers to investigate the mutation status of exon 19 and 21 of EGFR gene, respectively: 19HR01F: CTGGATCCCAGAAGGTGAGA; 19HR01R: GATTTCCTTGTTGGCTTTCG; 21HR01F: AGCCAGGAACGTACTGGTGA; 21HR01R: TGCCTCCTTCTGCATGGTAT. PCR conditions were 95°C for 5 min and 50 cycles of 95°C for 15 sec, 61°C >53°C (decreasing by 0.6°C with each cycle) for 20 sec, 72°C for 15 sec. For HRM analysis 95°C 1 min, 45°C 1 min and 61°C >95°C 25 sec. Finally 30 sec 30°C.

**Detection of KRAS mutations**

We used restriction fragment microfluidic-based detection (RFMD) PCR analysis to detect KRAS codon 12 mutations. First, genomic DNA was isolated from sections of paraffin-embedded tumors using QIAamp DNA FFPE Tissue Kit (Qiagen, Hilden, Germany). The PCR was performed with AmpliTaqGold PCR Master Mix (Applied Biosystems, Foster City, CA) using primers designed to introduce a BstnI restriction site in the products generated from the wild-type allele. The primer sequences were as follows: forward 5-GAATATAAACTTGTGGTAGTTGGACCT-3 and reverse 5-GGTCCTGCACCAGTAATATG-3. PCR conditions were 95°C for 10 minutes and 38 cycles of 95°C for 1 minute, 55°C for 1 minute, 72°C for 2 minutes, and finally 4 minutes at 72°C. The products obtained were then digested with BstnI (New England Biolabs, Beverly MA) at 60°C for 4 hours. This restriction enzyme digests the wild-type sequence but not the mutant thus identifying mutations in codon 12. All products were visualized on 3% agarose gels stained with ethidium bromide. We quantified the ratio of the mutant and wild-type products using Experion Automated Electrophoresis System (Bio-Rad Laboratories, Hercules, CA). The transitions in the mutant alleles were determined by direct sequence analysis using an Applied Biosystem 3130 Genetic analyzer (Applied Biosystems).

**Detection of HPV infection by PCR**

For the isolation of DNA, 4-5 pieces of 10-µm sections from the two samples were placed in sterile 2 ml Eppendorf tubes. Paraffin was removed by two washes in xylene followed by two washes in 96%v/v ethanol. Air-dried samples were digested overnight with 1 mg/ml Proteinase K in Tris-EDTA buffer (10 mM Tris, 1 mM EDTA pH=8.0). After high-speed centrifugation, the aqueous phase was separated and used as template solution. Each sample was assayed at least twice. The integrity of the DNA extracted from the archive tissues and the absence of inhibitors were controlled by subjecting the samples to PCR amplification with GH20 and PCO4 primers targeted to the human beta-globin gene.

Type-specific determination of HPVs was carried out by nested PCR. In the first round of the nested PCR, a pair of outer primers designed for a consensus sequence shared by the three most common high risk HPV types (16, 18, 33) was used to amplify E6 ORF sequence. One-microliter aliquots of the amplified products from the first round were used as templates for the next polymerase chain reaction. In this second round, primers specific for the HPV types 16, 18 and 33 were applied. Amplifications were performed with REDTaq ReadyMix (Sigma-Aldrich Co., St. Louis, Missouri, USA) in a PCR Express thermal cycler (Hybaid Ltd.-Thermo Electron Co.). A PCR reaction volume of 25 µl was chosen, containing 12.5 µl of 2× ReadyMix, 20 pmol of each primer and 1 μl of template solution. Amplicons were analyzed on 2% DNA-quality agarose gels (Pharmacia, Uppsala, Sweden) containing 0.5 g/ml ethidium bromide in Tris-acetate-EDTA buffer (TAE, pH 7.5), running at 5 V/cm (90 mA). PCR products were visualized and photographed using Kodak Image Station 4000MM gel-documentation system (Carestream Health Inc., Rochester, NY, USA).

In case none of the three HPV types could be found by the above method, an additional nested PCR assay using GP5+/GP6+ and MY9/MY11 primer pairs, targeted against L1 open reading frame, was attempted to detect the presence of HPV genome. These universal primer pairs recognize about 30 additional HPV types beside 16, 18 and 33.

**RT-PCR analysis of EGFR-ECD mRNA expression**

Total RNA was isolated from the *in vitro* growing PE/CA-PJ15 and PE/CA-PJ41 tumor cell lines using TRIzol^®^ Reagent (Invitrogen^TM^) according to the manufacturer’s instructions. Possible DNA contamination was eliminated using TURBO DNA-freeTM kit (Ambion^®^). For reverse transcription 1 μl of 10 mM dNTP mix (Finnzyme^®^) and 1μl of Random primer-oligo dT combination for a final concentration of 2.5 μM was added and used 2 μg of the purified total RNA. After incubating at 70 °C for 10 min, 2 μl of 10× M-MLV Reverse Transcriptase Buffer (SIGMA^®^), 1 μl of M-MLV Reverse Transcriptase (200 units/μl, SIGMA^®^), 0.5 μl RNase Inhibitor (40 units/μl, Promega^®^) and 6.5 μl DEPC-treated water was added for a final volume of 20 μl, than incubated at 37 °C for 50 min and 85 °C for 10 min. The occurrence of reverse transcription was checked by carrying out polymerase chain reaction with β-actin primers (GTG GGG CGC CCC AGG CAC CCA and CTC CTT AAT GTC ACG CAC GAT TTC) as a housekeeping gene. RNA of the same sample was used as negative control for detection of DNA contamination and DEPC-treated water was used as non-template control.

**Supplementary Table S1. Primer nucleotide sequences used in EGFR tyrosine kinase domain, KRAS, NRAS, HPV infection screening**

| Gene | Primer name | Sequence (5’ to 3’) | length |
| --- | --- | --- | --- |
| **KRAS** | KRASex2S | GAATATAAACTTGTGGTAGTTGGACCT | 144bp |
|  | KRASex2A | GGTCCTGCACCAGTAATATG |  |
|  | KRASex3S | CCTTCTCAGGATTCCTACAG | 175bp |
|  | KRASex3A | ATATCTTCAAATGATTTAGTATTATTTATGG |  |
|  | KRASex4S | TTTGAAAGATATTTGTGTTACTAATGAC | 228bp |
|  | KRASex4A | AGTGTTACTTACCTGTCTTGTC |  |
| **NRAS** | NRASex2S | TTGCTGGTGTGAAATGACTGAG | 191bp |
|  | NRASex2A | ATATGGGTAAAGATGATCCGACAAG |  |
|  | NRASex3S | AAACAAGTGGTTATAGATGGTGAAAC | 165bp |
|  | NRASex3A | GTAGAGGTTAATATCCGCAAATGAC |  |
|  | NRASex4S | CACTGTACCCAGCCTAATC | 278bp |
|  | NRASex4A | CTACCAGAGTTAATCAACTGATG |  |
| **HPV16E6** | HPV16E6S | AGTTACTGCGACGTGAGGTATA | 170 bp |
|  | HPV16E6A | GTATTGCTGTTCTAATGTTGTTCCA |  |
| **HPV18E6** | HPV18E6S | AGACAGTATTGGAACTTACAGAGG | 217bp |
|  | HPV18E6A | CACCGCAGGCACCTTATTAA |  |
| **HPV33E6** | HPV33E6S | TGGAGACAACTATACACAACATTGA | 160bp |
|  | HPV33E6A | TTAGATAAGAACCGCAAACACAGT |  |
| **HPV31E6** | HPV31E6S | ACTAAGATTGAATTGTGTCTACTGC | 311bp |
|  | HPV31E6A | CCTATGTTGTGGAATCGTCTCTTT |  |
| **HPV51E6** | HPV51S | CGAAGACAAGAGGGAAAGACCAC | 249bp |
|  | HPV51A | ACCATACACAGACCTGCTATAACG |  |
| EGFR tyrosine kinase domain screen | EGFR TK exon18 | GTGACCCTTGTCTCTGTGTT  CCAGGGACCTTACCTTATACA | 170bp |
|  | EGFR TK exon19 | GCCAGTTAACGTCTTCCTTCTCTC  CCACACAGCAAAGCAGAAACTCAC | 157bp |
|  | EGFR TK exon20 | CAGGAAGCCTACGTGATG  TGATTACCTTTGCGATCTG | 196bp |
|  | EGFR TK exon21 | TTCTTCCCATGATGATCTGT  TCCTTCTGCATGGTATTCTT | 199bp |

**Supplementary table S2:.Genetic test results on PE/CA-PJ15 and PE/CA-PJ41 HNSCC cell lines**

| Cell line | PE/CA-PJ15 | PE/CA-PJ41 |
| --- | --- | --- |
| EGFR tyrosine kinase domain screen | no alterations | no alterations |
| HPV 16, 18, 31, 33, 51 test | negative | negative |
| KRAS genotype test | wild type | wild type |
| NRAS genotype test | wild type | wild type |
| EGFR gene amplification test | no amplification | no amplification |
| EGFR N158N synonymous SNP | **homozygous mutant** | **heterozygous mutant** |
| EGFR T629T synonymous SNP | **heterozygous mutant** | wild type |
| EGFR R521K missense SNP | **heterozygous mutant** | wild type |

Both cell lines were found to be negative for HPV infection, KRAS, NRAS, or EGFR-TKD mutations. PE/CA-PJ15 harbors two mutant alleles of EGFR N158N synonymous, while is heterozygous at EGFR T629T synonymous and EGFR R521K missense mutation position. PE/CA-PJ41 cells were found to be heterozygous at EGFR N158N position, and homozygous wild type at both EGFR T629T and EGFR R521K. No EGFR gene amplification was measured in either of the two cell lines.
